# Prevalence of condylar morphological changes in individuals with class II malocclusion

**DOI:** 10.1590/1807-3107bor-2024.vol38.0060

**Published:** 2024-07-15

**Authors:** Daniela Fernandes Lobo Molica OLIVEIRA, Ellen Eduarda FERNANDES, Sergio Lúcio Pereira de Castro LOPES, Sigmar de Mello RODE, Wagner de OLIVEIRA, Ertty ERTTY, Mauricio de Almeida CARDOSO, An TIEN-LI, Fernanda MELOTI

**Affiliations:** (a) Institute of Science and Technology – UNESP, Department of Biosciences and Oral Diagnosis, São José dos Campos, SP, Brazil.; (b) Institute of Science and Technology – UNESP, Department of Diagnostics and Surgery, São José dos Campos, SP, Brazil.; (c) Institute of Science and Technology – UNESP, Department of Dental Materials and Prosthodontics, São José dos Campos, SP, Brazil.; (d) Faculdade São Leopoldo Mandic. Department of Orthodontics, Campinas, Brazil.; (e) University of Brasília – UNB, Department of Dentistry, Brasília, DF, Brazil.

**Keywords:** Cone-Beam Computed Tomography, Mandibular Condyle, Osteoarthritis

## Abstract

This observational, cross-sectional study with retrospective collection was aimed to evaluate the prevalence of morphological changes in mandibular condyles in individuals with class II malocclusion, classified according to different vertical growth patterns (brachyfacial, mesofacial, and dolichofacial), through cone beam computed tomography (CBCT). Seventy CBCT images (140 mandibular condyles) were selected from individuals without orthodontic treatment, of both sexes, aged between 25 and 50 years. No statistically significant differences were found between facial patterns; however, there was a higher relative prevalence of bone changes in dolichofacial individuals with flattening (62%), sclerosis (44%), and subchondral bone cyst (20%). Erosion and osteophytes prevailed in mesofacial (39%), and brachyfacial individuals (32%), respectively. Thus, there was no statistically significant difference in the prevalence of degenerative changes between the vertical skeletal patterns. Flattening was the most prevalent change, whereas subchondral bone cyst was the least prevalent among the three groups studied. The observational design of this study makes it possible to analyze image banks to verify the correlation of morphological changes in the temporomandibular joint in different facial patterns in patients with class II malocclusion. A limitation of the study is that clinical characteristics were not evaluated.

## Introduction

The temporomandibular joint (TMJ) is a mobile articulation with constant remodeling, where excessive mechanical stress may cause nonfunctional remodeling, thus altering its morphology.^1^ Orthodontic treatment, parafunction, macrotrauma, and unstable occlusion are the main mechanical factors that can initiate changes in the TMJ structures.^2,3^

Degenerative bone changes, also known as osteoarthrosis of the TMJ, are progressive and chronic, defined by gradual deterioration of the bone surface and characterized by the development of the following radiographic signs: flattening, osteophytes, subchondral bone cysts, bone sclerosis, and erosions.^4-6^

The prevalence of degenerative disorders is higher in older individuals^7-9^ and women,^8-10^ and differences have also been observed between the right and left sides.^9^ However, there is no consensus among evidence concerning age and sexual dimorphism.^11^

Condylar degenerative changes have been associated with the morphology of the articular eminence and roof of the fossa,^12^ clinical signs and symptoms of temporomandibular dysfunction,^4,6,13^ articular effusion, condylar position and vertical facial pattern,^14,15^ malocclusions,^16^ sagittal facial patterns,^5,11^ and condylar angulation in the axial plane.^17^

Considering the prevalence rates and associations, condylar degenerative changes may play a critical role and may interfere with the diagnosis and strategies of orthodontic treatment planning.^16,18^ Based on the surveyed data, it is not clear whether the prevalence rates with respect to the types of degeneration vary according to vertical skeletal patterns. Thus, this study evaluated the prevalence of different types of lesions in patients with class II malocclusion with different vertical patterns who already presented with condylar changes.

## Methods

### Ethical aspects

This cross-sectional observational study with retrospective collection was approved by the human subject’s ethics board of Faculdade São Leopoldo Mandic (CAAE 94068618.9.0000.5374) and was conducted in accordance with the Helsinki Declaration of 1975, as revised in 2013. The images were provided by the Solution3D Company.

### Sample

The research was performed using a database from a private diagnosis clinic. Cone-beam computed tomography (CBCT) images were selected from individuals with class II malocclusion (equal to or greater than half cusp), condylar changes, and the presence of all permanent teeth in the dental arches, except for the third molars.

The sample comprised CBCT recorded in patients for diagnosis from the year 2012 to 2018. The exclusion criteria were as follows: individuals not presenting skeletal asymmetries; crossbite; a history of fractures or polytrauma; syndromes; anomalies; tumors; ankylosis; developmental disorders; those who underwent orthognathic or TMJ surgeries, or those who previously underwent orthodontic treatment. The final sample was composed of the initial tomographic images of 70 individuals (140 mandibular condyles) with class II malocclusion of both sexes, with chronological age between 25 and 50 years. The sample was analyzed using the Dolphin Imaging® Software (Chatsworth, Califórnia, USA) to determine the facial pattern, according to Franco et al.,^19^ and subdivided into the following three groups: 11 brachyfacial, 14 mesofacial, and 45 dolichofacial individuals.

CBCT images were obtained from patients in maximum intercuspation, on an i-CAT tomography machine, with a voxel of 0.4 mm^3^, exposure time of 8.9 s, kilovoltage of 120 kVp, and alternate current of 36.9 mAs. The DICOM files of these tomographic examinations were submitted to CS 3D Imaging Software (Carestream Health Inc., Rochester, USA).

**Figure 1:**
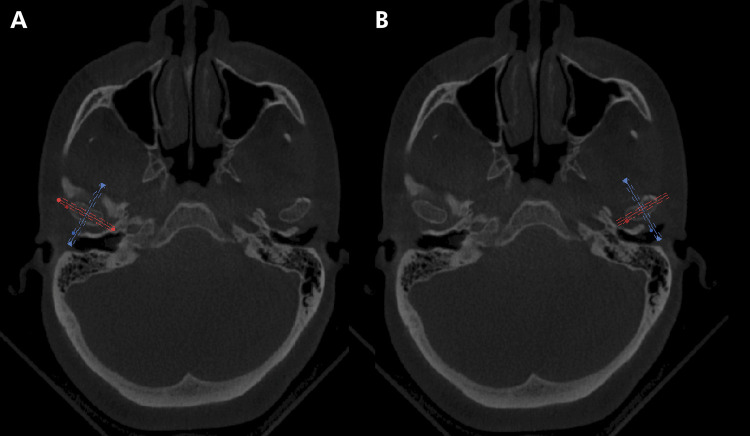
Achievement of temporomandibular joint (TMJ) images on the software CS 3D Imaging. The axial sections region is determined, identifying the greatest distance between the condylar poles on each side—right (A) and left (B). In this position, the most central point of the condyle is marked for achievement of the coronal and sagittal sections of the TMJs.

**Figure 2:**
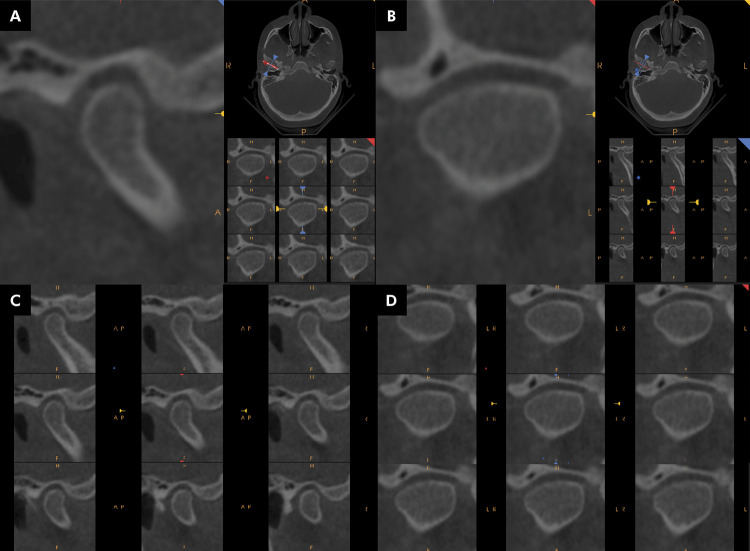
The most central sagittal (A) and coronal (B) sections are generated on each side—left and right—and five parasagittal (most medial and lateral) (C) and paracoronal (most anterior and posterior) (D) sections.

To standardize the slices to be analyzed, each TMJ was identified in the axial slices, and the long condylar axes were traced in the latero-medial direction (Figure 1), generating the parasagittal slices (0.4 mm) and perpendicular and paracoronal slices (0.4 mm), perpendicular and parallel to the axes, respectively. These cuts were then submitted to the evaluators. Brightness and contrast (window) were adjusted and enhancement filters were used to obtain better-quality images, thus simulating the real condition of the evaluation of examinations using images (Figure 2).

### Method for CBCT assessment

On the images obtained by the most central section, 10 images were obtained, and all were evaluated concerning the presence of changes in condylar morphology in the different vertical skeletal patterns. The condyle was considered to be affected by some degenerative change if at least one image presented characteristics suggesting this alteration.

The TMJ images were evaluated by a skilled professional and trained temporomandibular disorder (TMD) specialist. Two evaluations were performed, with a 30-day interval between the first and second evaluations, to verify the method error.

### Types of morphological changes of the mandibular condyles

**Figure 3:**
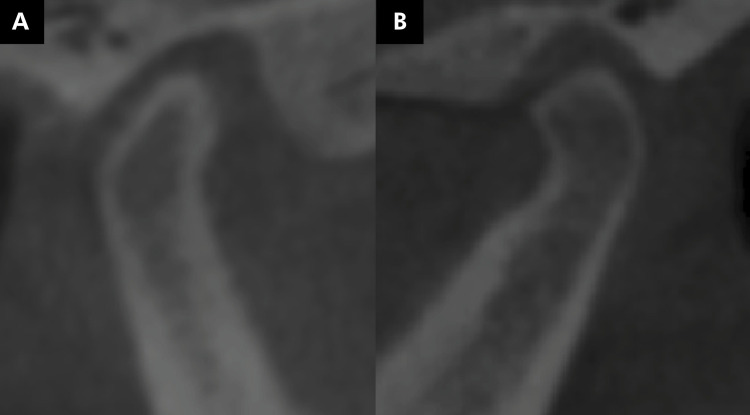
Image of the mandibular condyles obtained by cone-beam computed tomography, illustrating the presence of condylar flattening (loss of convexity or planing of the mandibular condyle) (A, B).

**Figure 4:**
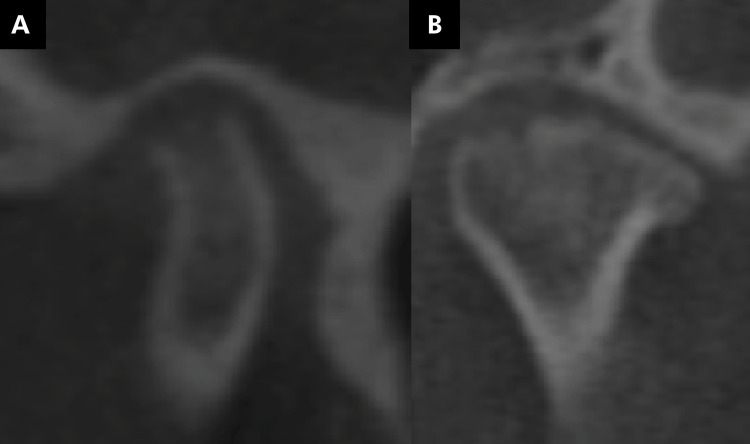
Tomographic image illustrating the presence of erosion (area with reduced density of cortical bone) (A, B).

**Figure 5:**
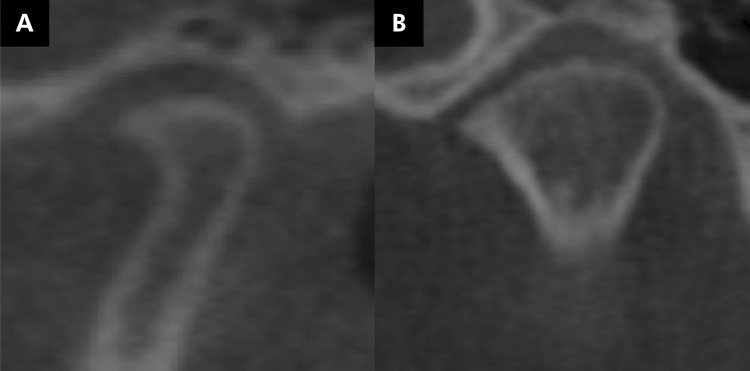
Tomographic image illustrating the presence of osteophyte (marginal hypertrophy with a sclerotic border and formation of bone tissue) (A, B).

**Figure 6:**
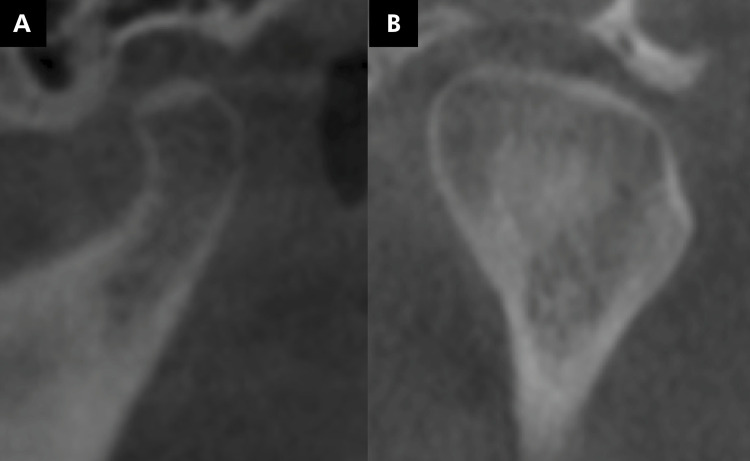
Tomographic image illustrating the presence of bone sclerosis (area of increased thickness of the cortical bone) (A, B).

**Figure 7:**
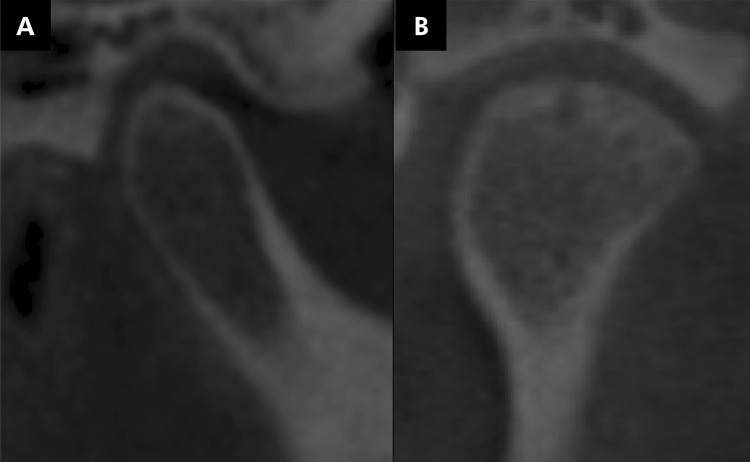
Tomographic image illustrating the presence of subchondral bone cyst (osteolytic area adjacent to the subcortical bone, without alteration of the cortical bone) (A, B).

The mandibular condyles were evaluated for the presence of changes in their morphology, flattening (Figure 3), erosion (Figure 4), osteophyte (Figure 5), bone sclerosis (Figure 6), and subchondral bone cyst (Figure 7), according to Kiliç et al. (2015).^6^

### Statistical methods

After collection, data were organized and statistically analyzed using the software SPSS version 24.0 (IBM Corp. Released 2016. IBM SPSS Statistics for Windows, Version 24.0. Armonk, NY: IBM Corp.). For age, the Shapiro–Wilk normality test was initially applied to evaluate the distribution of data. After observing that data presented a normal distribution, the ages of the different vertical pattern groups were compared using one-way analysis of variance (ANOVA).

Regarding the presence or absence of morphological changes in the different vertical skeletal patterns, the scores assigned by the examiner were organized into tables and compared using the chi-square test.

Finally, to analyze the method error, the intraexaminer agreement^20^ was analyzed using Cohen’s kappa test. The kappa coefficient was interpreted as follows: 0, poor agreement; 0.10–0.20, slight agreement; 0.21–0.40, fair agreement; 0.41–0.60, moderate agreement; 0.61–0.80, substantial agreement; 0.81–0.99, almost perfect agreement; and 1.00, perfect agreement.

## Results

Regarding the sample, the distribution did not present a statistically significant difference (Table 1). Dolichofacial individuals were the most prevalent (n = 90), followed by mesofacial (n = 28) and brachyfacial (n = 22) individuals (Table 1).

**Table 1:** Sample distribution according to the different vertical patterns and age.

Vertical pattern	n	Mean age	%
Brachyfacial	22	37 ± 7.8	15.71
Mesofacial	28	38.6 ± 7.6	20.00
Dolichofacial	90	35.7 ± 7.2	64.29
Total	140		100

There was no statistically significant difference between ages in the different groups.

In the absolute prevalence, without differentiation between vertical patterns, the greatest quantity of flattening and lowest of subchondral bone cysts were noted (Table 2). The prevalence of condylar morphological changes in the different vertical skeletal patterns is presented in Tables 3 and 4. No statistically significant differences were observed (at the 5% level) between the different vertical patterns.

**Table 2:** Prevalence of different morphological alterations in the total sample.

Pattern	Total sample (n = 140)			

	Presence		Absence	

	n	%	n	%
Osteophyte	35	25	105	75
Flattening	83	59	57	41
Erosion	43	31	97	69
Subchondral bone cyst	25	18	115	82
Sclerosis	59	42	81	58

**Table 3:** Prevalence of condylar morphological changes in three different types of vertical patterns in the total number of condyles (n=140).

Alteration	Brachyfacial (n = 22)				Dolichofacial (n =9 0)				Mesofacial (n = 28)				p-valor

	Absence		Presence		Absence		Presence		Absence		Presence		

Pattern	n	%	n	%	n	%	n	%	n	%	n	%	
Osteophyte	7	5	15	11	21	15	69	49	7	5	21	15	0,712
Flattening	11	8	11	8	56	40	34	24	16	11	12	9	0,56
Erosion	8	6	14	10	24	17	66	47	11	8	17	12	0,37
Subchondral bone cyst	4	3	18	13	18	13	72	51	3	2	25	18	0,533
Sclerosis	9	6	13	9	40	29	50	36	10	7	18	13	0,71

**Table 4:** Prevalence (in percentage) of condylar morphological changes for each facial vertical pattern.

Alteration	Brachyfacial (n = 22)				Dolichofacial (n = 9 0)				Mesofacial (n = 28)			

	Presence		Absence		Presence		Absence		Presence		Absence	

Pattern	n	%	n	%	n	%	n	%	n	%	n	%
Osteophyte	7	32	15	68	21	23	69	77	7	25	21	75
Flattening	11	50	11	50	56	62	34	38	16	57	12	43
Erosion	8	36	14	64	24	27	66	73	11	39	17	61
Subchondral bone cyst	4	18	18	82	18	20	72	80	3	11	25	89
Sclerosis	9	41	13	59	40	44	50	56	10	36	18	64

Regarding the intraexaminer error, the evaluation of osteophytes was in agreement k = 0.856, erosion was k = 0.917, and sclerosis was k = 0.912. For changes related to flattening, the agreement was k = 0.762, and for subchondral cyst k = 0.575.

## Discussion

Regarding the evaluation method used in this study, according to the level of agreement, the subchondral bone cyst presented lower agreement between the two intraexaminer evaluations, possibly because this morphological change was the most difficult to assess. The subjective classification parameters of condylar changes used in this study are not less valid or reproducible than others.^21^ According to Hill,^22^the higher the complexity of an evaluation method, the greater the chances of error caused by the examiner in the evaluations.

The age range distribution observed in this study was similar, presenting a mean of 35–38 years, without statistically significant differences between the three skeletal patterns. Pontual et al.^23^ highlighted that during the growth period of individuals, between 3 and 20 years, the mandibular condyles tend to present few physiological changes. The TMJ undergoes constant bone remodeling (renewal of cellular and extracellular matrix); thus, excessive mechanical stresses may cause nonfunctional remodeling, altering its morphology.^4^ With increasing age, progression and worsening of bone changes in the mandibular condyles are noted.^23^

Several studies have investigated joint disorders in patients with class II malocclusion.^3,5,10,14,16,24^ Katsavrias^16^observed significant morphological changes in the TMJ, concerning the anteroposterior dimension of the articular fossa, height and inclination of the eminence, and morphology of the mandibular ramus.^18^ Fraga et al.^24^ observed greater decentralization of the mandibular condyles in patients with this type of malocclusion. Using CBCT, Dygas et al.^3^ studied the relationship between degenerative changes in the TMJ, craniofacial morphology, and malocclusion. They observed that more than one type of degeneration occurred in approximately 6% of condyles. The most common change was faceting in 52.3% of individuals, and approximately one-third of the articular eminences showed degenerative changes. In skeletal classes I and III, condylar faceting was the most prevalent degeneration, while in class II osteophytes and faceting were detected.

Some studies have demonstrated that the condylar position and morphology are more related to the vertical skeletal pattern and observed a higher prevalence of internal condylar changes in patients with a hyperdivergent pattern. The increased articular space in hyperdivergent individuals, caused by displacement of the articular disc, has already been observed, evidencing that when the articular disc altered its position between the cranial base and condyle, TMDs and morphological changes were observed.^15,25^ Hyperdivergent patients with class II malocclusion present with an increased frequency of articular disc displacement, consequently presenting condylar degenerative disorders.^15,26^ In these individuals, condylar changes compensatory to the articular disc displacement occur during the growth period.^27^ There is a significant correlation between mandibular morphology and changes in condylar morphology because abnormal mandibular growth may influence the occurrence of these changes.^28^ The relationship between TMJ and occlusion has benefited from studies using CBCT, which allows more precise assessments between the condyle and fossa, integrity of the articular surfaces, and, more recently, condylar volume.^29^

Sampling was performed for convenience; the exclusion criteria restricted the sample to nontreated, nonsyndromic, and nonasymmetric individuals. For ethical reasons, tomographic images were difficult to obtain when all the exclusion criteria were applied. Therefore, this cannot be regarded as an epidemiological study.

The sample selected for this study evidenced a greater number of dolichofacial individuals than mesofacial and brachyfacial individuals. This disproportion was related to the fact that the vertical skeletal pattern was casually selected after the separation of class II malocclusions, with changes in condylar morphology. To observe the types of condylar changes, all individuals should present alterations, and class II malocclusion presents most changes because of the mandibular morphology of these individuals.^10,28^ Therefore, to achieve an actual comparison of the findings, it was necessary to use the relative frequency because the absolute frequency would cause bias owing to the unbalanced sample size between the different vertical patterns.

In the present study, the prevalence of different types of degenerative changes was not significantly different between the different vertical skeletal patterns; however, some interesting tendencies may be indicated.

Flattening was the most prevalent degenerative change, corroborating the results of previous studies.^6,8,9,30-32^ This common evidence is probably because flattening is a morphological change within normality as it is a precursor of the degenerative processes of articular diseases, being part of the physiological remodeling of articulation aging.^7,23,33^

Subchondral bone cyst was the least prevalent alteration in the three study groups, which also corroborated other studies.^6,30^

The most prevalent alterations in the dolichofacial group were flattening (62%) and bone sclerosis (44%). When comparing the relative frequency with other groups, the dolichofacial group appeared to present a higher prevalence of the following four types of changes: flattening (62%), bone sclerosis (44%), and subchondral bone cyst (20%). The increased percentage of degeneration may have been observed as a consequence of the mandibular morphology of these individuals, whose main characteristic is the presence of differentiated muscular activity, with consequent overload of intra-articular pressure.^34^ The change in the condylar load is the basic mechanism of condylar changes because the joints do not resist the new force vectors and undergo biochemical, cellular, and functional changes.^34^ However, mandibular morphology produces changes in intra-articular pressure because other aspects may be involved. Condylar changes may occur due to several etiological factors related to occlusion (macro- and microtraumas, repetitive traumas, parafunctional habits, and tooth losses); systemic changes; adverse life events (such as stress); and craniofacial morphology.^27^ The etiological factors may be related to some types of trauma such as functional overload, joint laxity, masticatory muscle spasm, and increased attrition between the mobile parts.^2,34,35^ Several studies^5,16,27^ have observed articular disorders in dolichofacial individuals with class II malocclusion, considering that the pathological processes initiate on the periphery toward the joint center.^36^

For the brachyfacial group, the most prevalent alterations were flattening (50%), bone sclerosis (41%), and erosion (36%). Additionally, concerning the other groups, there was a higher relative frequency of osteophytes (32%) in brachyfacial individuals. This vertical skeletal pattern is characterized by greater muscular function of the masseter and lateral pterygoid. The pterygoid muscle in these individuals presents stronger traction of the mandibular condyle,^5,28^ which is associated with degeneration of the cartilage that protects the bone and tends to form this bone surface (osteophyte) in an attempt to better afford the force loads.^34^ An osteophyte is a sign that the condyle is adapting or has adapted to past degenerative changes.^4^

For individuals with a mesofacial vertical skeletal pattern, the most prevalent changes were flattening (57%) and erosion (39%). There was a greater relative frequency of erosion in this group than that in the other groups, being a change that represents the initial stage of the degenerative bone process.^6^

When the prevalence of lesions was compared between groups, flattening, sclerosis, and subchondral bone cysts were greater in dolichofacial individuals, erosion was more common in mesofacial individuals, and osteophytes were more common in brachyfacial individuals. The occurrence and significance of these facts should be elucidated in future studies.

## Conclusion

Despite the limitations of this study, based on these data, it could be concluded that there was no statistically significant difference in the prevalence of degenerative changes between the different groups of vertical skeletal patterns. The occurrence of flattening was the most prevalent, whereas subchondral bone cysts were less prevalent in the three study groups.
